# Efficacy of combining soothing moisturizing repairing cream with glucocorticoids in the treatment of moderate atopic dermatitis in pediatric patients: A prospective observational cohort study

**DOI:** 10.1097/MD.0000000000047171

**Published:** 2026-01-30

**Authors:** Zhiwei Guan, Yangyang Lin, Ying Wang, Lixin Chen, Qinfeng Li

**Affiliations:** a Department of Dermatology, Tianjin Children’s Hospital, Tianjin University Children’s Hospital, Tianjin, China.

**Keywords:** atopic dermatitis, cohort study, glucocorticoids, moisturizing repairing cream, pediatric

## Abstract

Glucocorticoids are the most commonly used drugs for the treatment of atopic dermatitis (AD) in children, this study aimed to explore the therapeutic efficacy of combining moisturizers and glucocorticoids in the treatment of moderate AD in pediatric patients. Patients aged 6 to 12 years with moderate AD were involved. The control group received fluticasone propionate cream twice daily, and the experimental group received soothing moisturizing repairing cream combined to the fluticasone. Both groups treated continuously for most 4 weeks until the Symptom Score Reduction Index ≥90% and then entered a 2-week remission phase. Clinical outcomes, fluticasone dosage, Children’s Dermatology Life Quality Index, AD recurrence rates, remission duration, and adverse events were compared at the end of the remission period. A total of 54 patients completed the follow-up, with 25 in the control and 29 in the experimental group. The overall clinical efficiency in the experimental group (100%, 95% confidence interval: 88.1–100.0%) was significantly higher than in the control group (84%, 95% confidence interval: 63.9–95.5%) with *P* = .040. Both groups showed a decrease in Children’s Dermatology Life Quality Index. The recurrence rate was remarkably lower, and the remission duration was longer in the experimental group (both *P* < .05). Combining soothing moisturizing repairing cream with fluticasone cream has good clinical efficacy in the treatment of moderate AD in children. This combination therapy can effectively reduce the risk of relapses during the remission period, improve clinical symptoms, and improve quality of life in children with AD.

## 1. Introduction

Atopic dermatitis (AD) is one of the most prevalent chronic and relapsing inflammatory skin diseases in children, characterized by eczematous lesions and intense pruritus. AD is a common disease among pediatric patients, impacting up to 15% to 20% of children worldwide.^[1]^ In China, the prevalence of AD varies, affecting about 8% of children aged 3 to 6 years,^[2]^ with rates increasing to 20% in children from birth to 14 years old.^[3]^ According to the epidemiological studies in 2014, the prevalence of AD was 12.94% among children aged 1 to 7 years in China.^[4]^ This condition predominantly affects children, leading to intense itching, localized skin hypertrophy, and hyperpigmentation. Without proactive treatment, it may have adverse effects on the growth, development, and physical and mental health of children, thereby profoundly impacting their quality of life.^[5–8]^

The pathogenesis of AD is influenced by both genetic and environmental factors.^[9]^ The guidelines for the diagnosis and treatment of AD suggest that impaired skin barrier function and inflammatory response are important factors in the occurrence of AD.^[10]^ Several studies have pointed out that dry skin plays pivotal roles in the onset of AD.^[11]^ Topical glucocorticoids are considered the first-line treatment for AD in clinical practice. However, long-term usage can lead to adverse effects such as skin atrophy and telangiectasia.

Fluticasone propionate (FP) is a synthetic fluorinated corticosteroid known for its strong anti-inflammatory effects and 0.05% FP cream has shown a high therapeutic index and a favorable risk-benefit profile.^[12,13]^ Friedlander reported that FP had efficacy in the treatment of moderate to severe AD in children aged 3 months and older.^[14]^ Topical FP is a potent corticosteroid with its low rate of percutaneous absorption, which in turn leads to a low potential for systemic toxicity.^[15,16]^ Glazenburg study demonstrated that intermittent FP treatment resulted in less severe AD and a significant reduction in the risk of further relapses in children with moderate to severe AD.^[17]^

In early 2014 and 2020, Chinese guidelines for AD management recommend the application of emollients in both the acute and maintenance phases.^[18,19]^ It has been widely believed that the consensus has shifted towards considering hydration and lubrication with topical moisturizers and emollients as fundamental in treating AD in infants and young children.^[20]^ When combined with corticosteroids, this can achieve better clinical effects. Multiple studies also suggested the use of topical emollients to reduce the frequency and intensity of AD episodes.^[21–23]^ John research^[24]^ showed that active use of fluticasone twice a week in addition to daily use of emollients in adults can significantly prolong the time to recurrence, reduce the number of recurrences, and reduce the total amount of hormones used. Zhang et al also stated the combination of moisturizing cream and desonide in the treatment of AD in children is highly effective and safe compared to desonide only group.^[25]^ The American Academy of Dermatology guidelines also clearly recommend that emollients should be used as the basis of treatment for all AD patients and should be used concurrently with anti-inflammatory treatment.^[26]^ A prospective study also found that consistent moisturizer application after washing was associated with decreased severity of AD.^[27]^ A study on a ceramide-precursor-based moisturizer also found that two-thirds of AD patients showed significant improvements in objective Scoring of Atopic Dermatitis (SCORAD) scores and skin hydration.^[28]^

This cohort study was designed to evaluate the therapeutic efficacy of soothing moisturizing repairing cream in moderate AD and its impact on the dosage of topical glucocorticoids and the recurrence rate.

## 2. Methods

### 2.1. Study design

We evaluated the overall response of glucocorticoids in pediatric patients with moderate AD (with SCORAD scores between 25 and 50),^[19]^ which was 40% and expected an overall response of 75% for the combination treatment. The planned sample size was 56 patients based on Simon optimal two-stage design (with a two-sided α value of 5% and power of 80%). Considering 10% potential withdrawals in each group, we aim to include at least 70 patients. Therefore, 70 children aged 6 to 12 years with moderate AD from the Department of Dermatology at Tianjin Children’s Hospital between November 2022 and March 2023 were involved. The ethical statement was approved by the Ethics Committees of Tianjin Children’s Hospital (No. KY2020-31), and this study was conducted in accordance with the Declaration of Helsinki of 2008 and Good Clinical Practice guidelines. Written informed consent was obtained from the legally acceptable representatives of the patients.

**Figure 1.**
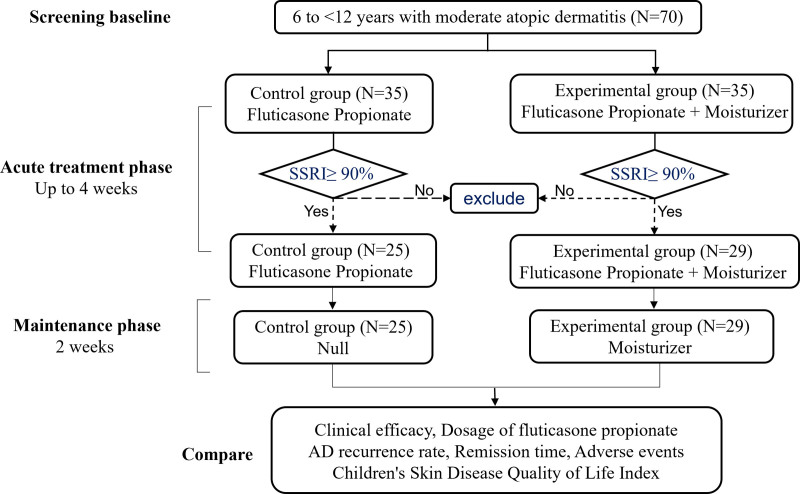
Study workflow.

This study adopted the method of consecutive enrollment to recruit patients, and eligible patients were assigned to the control group (n = 35) and experimental group (n = 35). The study workflow can be seen in Figure 1, during the treatment phase, control group patients received only topical application of 0.05% fluticasone propionate cream twice daily, while the experimental group patients applied soothing moisturizing repairing cream over the whole body twice daily, before the application of fluticasone cream. Both groups were treated continuously for 1 to 4 weeks until the affected skin achieved healing (Symptom Score Reduction Index [SSRI] ≥ 90%). Then entered 2-week maintenance phase, during which the control group discontinued the use of fluticasone, while the experimental group continued applying the moisturizer twice daily. Throughout the study, the patients’ guardians consistently maintained a “Medication Diary” using the fingertip unit method to record daily usage of the 0.05% fluticasone propionate cream. Scheduled visits were performed on day 7, day 14, day 21, and day 28 (±2 days) during treatment until lesions had fully healed. The relapse rates were recorded at the end of the remission phase. Clinical efficacy, fluticasone usage, AD recurrence rate, remission duration, and adverse events were compared between 2 groups. The Children’s Dermatology Life Quality Index (CDLQI)^[29]^ was carried out using 1 week before the start of treatment and on the first day of the maintenance phase.

### 2.2. Inclusion, exclusion, and termination criteria

Inclusion criteria: patients aged 6 to 12 years, with SCORAD scores^[30]^ between 25 and 50, with informed consent from guardians.

Exclusion criteria: participation in any other clinical trial within the past 3 months or current participation; use of systemic glucocorticoids or antihistamines within 4 weeks prior to study enrollment; a history of severe systemic diseases (e.g., cardiovascular, hepatic, renal) or any other clinically unstable condition that would compromise patient safety or interfere with study participation.

*Termination criteria*: lesions did not achieve remission within 4 weeks; withdrawal of informed consent by the guardians; violation of the clinical protocol, poor compliance leading to voluntary exit; development of severe adverse reactions requiring treatment termination.

### 2.3. Efficacy indicators

Therapeutic efficacy was assessed by the SCORAD index, the amount of 0.05% fluticasone propionate cream used, the CDLQI, relapse rates and remission duration. The SSRI is the percentage reduction in SCORAD. We defined the treatment outcomes based on the SSRI as follows: recovery (SSRI ≥ 90%), apparent effective (SSRI 60–89%), effective (SSRI 20–59%), and inefficient (SSRI < 20%). Relapse is defined as the reappearance of lesions with a SCORAD score ≥10% of the baseline SCORAD score in patients who had reached the maintenance phase. The relapse rate was calculated as the percentage of relapsed patients who had entered the maintenance phase. Remission duration was measured as the time from entering the maintenance phase to the occurrence of a relapse.

### 2.4. Safety assessment

Adverse reactions associated with the use of soothing moisturizing repairing cream were evaluated for safety.

### 2.5. Statistical analysis

Data was stored and managed using the Microsoft Access 2010 database, statistical analysis was performed using SPSS software (v27.0; IBM Corp., Armonk). Chi-squared tests, adopting χ2 test, Fisher exact probability method, group *t* test, paired *t* test, Mann–Whitney *U* test, Wilcoxon paired sign rank sum test were used to analyze the difference. The log-rank test was used to assess differences between 2 groups, and the Kaplan–Meier product limit method was used to estimate the distribution of time to AD relapse during the maintenance phase. *P*-value <.05 considered statistically significant.

## 3. Results

### 3.1. Study population and patient disposition

A total of 70 patients were initially enrolled and equally (n = 35 per group) to either twice-daily 0.05% FP cream alone (control group) or to the same FP regimen combined with soothing moisturizing repairing cream (experimental group). Sixteen patients (22.9%) were lost to follow-up, experienced inadequate efficacy or violated the protocol. Finally, 54 patients (77%) entered the maintenance phase, with 25 (71%) in the control group and 29 (83%) in the experimental group. The demographic and baseline information between the 2 groups showed no statistically significant differences (Table S1, Supplemental Digital Content, https://links.lww.com/MD/R235). At baseline, the mean SCORAD score was 36.52 ± 4.45 in the experimental group and 36.53 ± 4.78 in the control group, with no significant difference between the groups (*P* = .995). Similarly, the mean CDLQI score was 8.05 ± 1.93 and 8.32 ± 2.64 in the experimental and control groups, respectively, and this difference was also not statistically significant (*P* = .526).

### 3.2. Clinical efficiency

We assessed the number of recovery (SSRI ≥ 90%), apparent effective (SSRI 60–89%), effective (SSRI 20–59%), and inefficient (SSRI < 20%) cases at 7, 14, and 21 days after treatment. The overall rate of clinical effectiveness (recovery + apparent effective) in experimental group was 75.86% (95% confidence interval [CI], 56.5–89.7%), 86.21% (95% CI, 68.3–96.1%), and 100% (95% CI, 88.1–100.0%) at 7, 14, and 21 days, respectively. Meanwhile, the efficiency rates in control group were 44% (95% CI, 24.4–65.1%), 56% (95% CI, 34.9–75.6%), and 84% (95% CI, 63.9–95.5%) at 7, 14, and 21 days, respectively. The clinical efficacy at each assessment point was significantly higher in the experimental group compared to the control group with *P* = .017, 0.014, and 0.040 respectively (Table 1).

**Table 1:** Clinical efficiency between experimental and control group.

Time	Group (n)	Recovery	Apparent effective		Effective		Inefficient	Efficiency (%) 95% CI	χ2	*P*
7 d after treatment	Exp (29)	6	16	6		1		22 (75.9%) 56.5–89.7%	5.7.4	.017
	Con (25)	2	9	10		4		11 (44.0%) 24.4–65.1%		
14 d after treatment	Exp (29)	18	7	4		0		25 (86.2%) 68.3–96.1%	6.11	.014
	Con (25)	10	4	10		1		14 (56.0%) 34.9–75.6%		
21 d after treatment	Exp (29)	24	5	0		0		29 (100.0%) 88.1–100.0%	Fisher	.040
	Con (25)	17	4	4		0		21 (84.0%) 63.9–95.5%		

Con = control, d = day, exp = experimental.

### 3.3. Usage of fluticasone propionate

At 7, 14, 21, and 28 days after treatment, the mean cumulative dosage of 0.05% FP (grams) used in the experimental group was 17.95 ± 4.53, 29.02 ± 7.64, 41.50 ± 5.07, and 46.20 ± 2.39, respectively, and the usage in control group was 23.02 ± 4.61, 36.54 ± 6.64, 47.70 ± 5.97, and 54.38 ± 7.76 (Table 2). At all 4 time points, the usage in the experimental group was significantly lower than that in the control group.

**Table 2:** Comparison the dosage of fluticasone propionate between 2 groups.

Time	Experimental group	Control group	*T*/*z*	*P*
7 d after treatment	17.95 ± 4.53	23.02 ± 4.61	‐4.068	<.001
14 d after treatment	29.02 ± 7.64	36.54 ± 6.64	‐3.564	.001
21 d after treatment	41.50 ± 5.07	47.70 ± 5.97	‐2.782	.010
28 d after treatment	46.20 ± 2.39	54.38 ± 7.76	‐2.349	.019

### 3.4. Children’s Dermatology Life Quality Index

The subcategories of CDLQI, including symptoms and feelings, social interactions, daily activities, focus, sleep and treatment effects, were evaluated. Significant improvements were observed in the experimental group across all dimensions with *P* < .01 (Table 3).

**Table 3:** Comparison of CDLQI score in subcategories between 2 groups.

Group	Symptoms and feelings	Social interactions	Daily activities	Focus	Sleep	Treatment effects
Experimental	0.60 ± 0.33	0.46 ± 0.30	0.47 ± 0.34	0.24 ± 0.31	0.59 ± 0.31	0.61 ± 0.34
Control	1.09 ± 0.45	0.76 ± 0.41	0.80 ± 0.36	0.55 ± 0.14	1.02 ± 0.44	1.12 ± 0.36
*T*/*z*	‐4.626	‐3.053	‐3.518	4.239	‐4.132	‐5.306
*P*	<.001	.004	.001	<.001	<.001	<.001

CDLQI = Children’s Dermatology Life Quality Index.

### 3.5. Relapse rate and duration of remission

**Figure 2.**
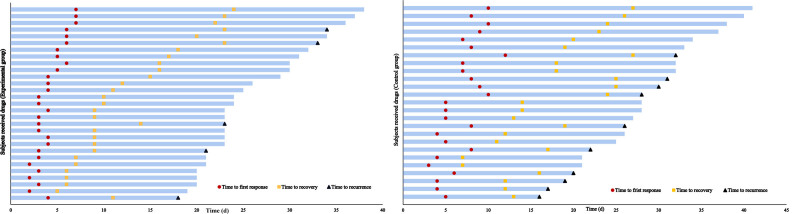
Response time of each subjects in experimental and control group.

During the maintenance phase, the number of patients who experienced AD relapses was 10 (40%) in control group, while in the experimental group there were only 5 patients (17.2%). The median time to first response in experimental group was 4 days (95% CI, 3.3–4.7), while in control group was 7 days (95% CI, 5.4–8.6). The median recovery time in the experimental group was 11 days (95% CI, 8.4–13.6), and in the control group was 18 days (95% CI, 14.8–21.2). Both the response and recovery time in the experimental group was significantly lower than that in the control group with *P* = .000 and .004 respectively. At the end of the maintenance phase, the duration of remission in the experimental group (13.79 ± 2.37 days) was significantly longer than that in the control group (10.44 ± 4.52 days) (*P* = .004). These findings are presented in Figure 2A for the experimental group and in Figure 2B for the control group.

**Figure 3.**
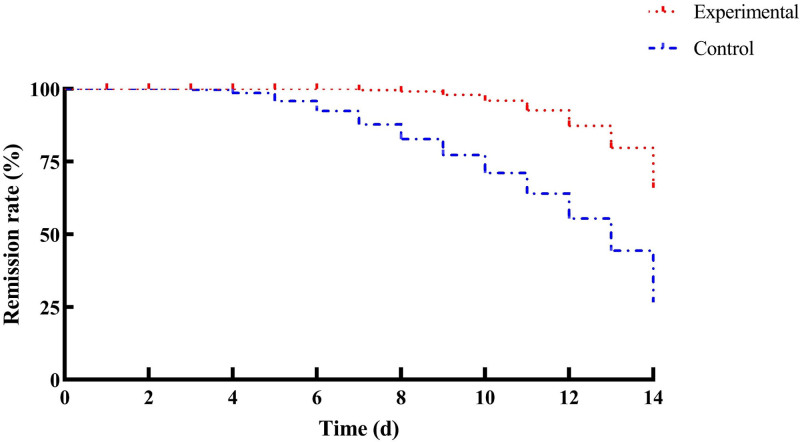
Remission rate between 2 groups.

According to the Cox model, the relative hazard ratio value was 0.334 (95% CI, 0.114–0.980), implying that the remission rate of AD maintenance in the experimental group was better than that in control group with *P* = .046. The Kaplan–Meier survival plot of time to the first relapse of AD during maintenance phase by treatment was shown in Figure 3.

### 3.6. Safety assessment

Throughout the observation period, no adverse reactions were reported in both groups.

## 4. Discussion

The pathogenesis of AD is not yet fully understood, and the disruption of the skin barrier would leads to increased trans epidermal water loss, skin infection, and more exposure to irritants and allergens.^[31]^ Dysfunctional skin barriers result in skin dryness, which is a primary clinical feature of AD, daily full-body application of moisturizers remains the cornerstone of AD management.^[32]^

Emollients are widely acknowledged as the foundation of AD therapy and the basis of long-term management.^[33]^ Both the 2018 and 2022 European AD guidelines recommend the use of emollients containing anti-inflammatory active substances as emollient plus, which plays an important role in the basic treatment of AD.^[10,20]^ Moisturizers have been widely accepted as the cornerstone of AD therapy.^[26,34–36]^ Several studies have demonstrated, medical skincare products, considered special cosmetics with therapeutic effects, have been proven to be able to repair skin barriers and aid in the treatment of AD.^[21–23,37]^

The investigational moisturizer comprises 4 key active ingredients: 4-tert-butylcyclohexanol, triterpenoids and sterol/triterpene ester, carbohydrate isomers, and licorice chalcone A. It can rapidly alleviate itching, provide long-lasting moisturization, and exhibit multifaceted anti-inflammatory effects, effectively restoring the fragile skin barrier in young children. 4-Tert-butylcyclohexanol is an effective transient receptor potential vanilloid 1 antagonist that can quick reducing itching, stinging, and burning sensations, it can reduce the side effects of glucocorticoids and shorten the duration.^[38]^ Triterpenoids and sterols/triterpene esters are known for skin-soothing properties, reinforcement skin’s barrier and enhancement of skin barrier function. Carbohydrate isomers can bind to lysine in keratin of keratinocytes, promoting short-term hydration and moisture retention in the stratum corneum. Glycyrrhetinic acid chalcone A, serves as an inhibitor of phosphodiesterase-4 can effectively reduce the release of pro-inflammatory cytokines, and inhibit the release of inflammatory mediators or cytokines such as IL-1β and IL-6, playing an important anti-inflammatory role.

Our study demonstrated that active use of emollients with repair functions after controlling acute AD lesions can reduce the risk of AD recurrence. After 2 weeks of AD maintenance, the AD maintenance remission rate was higher in the experimental group. During AD maintenance, the first recurrence time in the experimental group was delayed compared to the control group. Therefore, consistent use of moisturizer at the maintenance period can effectively reduce the recurrence of AD inflammation and extend the time before recurrence. Several studies have indicated that regular emollient application has a short- and long-term steroid sparing effect in mild to moderate AD.^[36,39]^ International guidelines recommend moisturizing the affected and unaffected skin twice daily as a fundamental treatment for AD. For moderate to severe cases, it should act as supplementary therapy to improve the skin barrier function and delay relapse.^[40]^ In a clinical trial, 124 newborns with a family history of AD at 6 months of age, only 22% of newborns receiving daily moisturizing therapy developed AD, while the control group without moisturizing therapy had a 43% incidence of AD. This indicated a 50% relative risk reduction, thus supporting the effectiveness of moisturizers in the primary prevention of AD.^[41]^

The safety assessments in our study also indicated that combining FP with emollient during the acute phase and using emollient during the maintenance phase did not cause serious adverse events. AD exerts a significant influence on the quality of life for both affected children and their families. Our research reveals that, after the treatment, the quality of life in various aspects improved significantly between 2 groups. Notably, the degree of improvement was markedly superior in the experimental group. In terms of CDLQI subcategories, the scores for symptoms and feelings, focus, sleep and treatment effects in experimental group were lower than control group.

The limitations of our study include the small sample size and the potential for further improvement with a longer maintenance and follow-up phase. As a single-center study, our findings may be influenced by local patient demographics and specific treatment protocols, potentially limiting their generalizability to other settings. Future large-scale prospective studies are deemed needed.

In conclusion, the soothing moisturizing repairing cream in our study exhibits promising clinical efficacy and safety in the adjunctive treatment of moderate pediatric AD. It can effectively reduce the dosage of topical corticosteroids, significantly lower the risk of relapses during remission periods, improve patient outcomes, and enhance the overall quality of life. Therefore, its application is recommended for broader clinical utilization and inclusion in the daily skincare routine for children with AD.

## Author contributions

**Conceptualization:** Zhiwei Guan, Yangyang Lin.

**Data curation:** Ying Wang.

**Formal analysis:** Zhiwei Guan.

**Investigation:** Lixin Chen.

**Methodology:** Lixin Chen.

**Writing – original draft:** Qinfeng Li.

**Writing – review & editing:** Qinfeng Li.

## Supplementary Material



## References

[1] Asher MI Montefort S Björkstén B. Worldwide time trends in the prevalence of symptoms of asthma, allergic rhinoconjunctivitis, and eczema in childhood: ISAAC phases one and three repeat multicountry cross-sectional surveys. Lancet. 2006;368:733–43. 16935684 10.1016/S0140-6736(06)69283-0

[2] Xu F Yan S Li F. Prevalence of childhood atopic dermatitis: an urban and rural community-based study in Shanghai, China. PLoS One. 2012;7:e36174. 22563481 10.1371/journal.pone.0036174 PMC3341360

[3] Zhao J Bai J Shen KL. Questionnaire-based survey of allergic diseases among children aged 0–14 years in the downtown of Beijing, Chongqing and Guangzhou [in Chinese]. Zhonghua Er Ke Za Zhi. 2011;49:740–4. 22321178

[4] Guo Y Li P Tang J. Prevalence of atopic dermatitis in Chinese children aged 1–7 ys. Sci Rep. 2016;6:29751. 27432148 10.1038/srep29751 PMC4949439

[5] Guttman-Yassky E Renert-Yuval Y Brunner PM. Atopic dermatitis. Lancet. 2025;405:583–96. 39955121 10.1016/S0140-6736(24)02519-4

[6] Wang Q Liu L Gao S Su S. Guidelines for the management of atopic dermatitis in children: a systematic review. Int Arch Allergy Immunol. 2023;184:132–41. 36323240 10.1159/000527007

[7] Boguniewicz M Leung DYM. Atopic dermatitis: a disease of altered skin barrier and immune dysregulation. Immunol Rev. 2011;242:233–46. 21682749 10.1111/j.1600-065X.2011.01027.x PMC3122139

[8] Lewis-Jones S. Quality of life and childhood atopic dermatitis: the misery of living with childhood eczema. Int J Clin Pract. 2006;60:984–92. 16893440 10.1111/j.1742-1241.2006.01047.x

[9] Liang Y Chang C Lu Q. The genetics and epigenetics of atopic dermatitis-filaggrin and other polymorphisms. Clin Rev Allergy Immunol. 2016;51:315–28. 26385242 10.1007/s12016-015-8508-5

[10] Wollenberg A Kinberger M Arents B. European guideline (EuroGuiDerm) on atopic eczema-part II: non-systemic treatments and treatment recommendations for special AE patient populations. J Eur Acad Dermatol Venereol. 2022;36:1904–26. 36056736 10.1111/jdv.18429

[11] Yang G Seok JK Kang HC Cho YY Lee HS Lee JY. Skin barrier abnormalities and immune dysfunction in atopic dermatitis. Int J Mol Sci . 2020;21:2867. 32326002 10.3390/ijms21082867 PMC7215310

[12] Korting HC Schöllmann C. Topical fluticasone propionate: intervention and maintenance treatment options of atopic dermatitis based on a high therapeutic index. J Eur Acad Dermatol Venereol. 2012;26:133–40. 21977914 10.1111/j.1468-3083.2011.04195.x

[13] Brazzini B Pimpinelli N. New and established topical corticosteroids in dermatology: clinical pharmacology and therapeutic use. Am J Clin Dermatol. 2002;3:47–58. 11817968 10.2165/00128071-200203010-00005

[14] Friedlander SF Hebert AA Allen DB; Fluticasone Pediatrics Safety Study Group. Safety of fluticasone propionate cream 0.05% for the treatment of severe and extensive atopic dermatitis in children as young as 3 months. J Am Acad Dermatol. 2002;46:387–93. 11862174 10.1067/mjd.2002.118337

[15] Spencer CM Wiseman LR. Topical fluticasone propionate: a review of its pharmacological properties and therapeutic use in the treatment of dermatological disorders. BioDrugs. 1997;7:318–34. 18020489 10.2165/00063030-199707040-00006

[16] Thalén A Brattsand R Andersson PH. Development of glucocorticosteroids with enhanced ratio between topical and systemic effects. Acta Derm Venereol Suppl (Stockh). 1989;151:11–9; discussion 47. 2624062

[17] Glazenburg EJ Wolkerstorfer A Gerretsen AL Mulder PGH Oranje AP. Efficacy and safety of fluticasone propionate 0.005% ointment in the long-term maintenance treatment of children with atopic dermatitis: differences between boys and girls? Pediatr Allergy Immunol. 2009;20:59–66. 18298423 10.1111/j.1399-3038.2008.00735.x

[18] Gu H Zhang JZ; Chinese Society of Dermatology. Guidelines for the diagnosis and treatment of atopic dermatitis in China (2014) [in Chinese]. Chin J Dermatol. 2014;47:511–4.

[19] Gu H Zhang JZ; Chinese Society of Dermatology. Chinese guideline for diagnosis and treatment of atopic dermatitis (2020) [in Chinese]. Chin J Dermatol. 2020;53:81–8.

[20] Wollenberg A Barbarot S Bieber T. Consensus-based European guidelines for treatment of atopic eczema (atopic dermatitis) in adults and children: part I. J Eur Acad Dermatol Venereol. 2018;32:657–82. 29676534 10.1111/jdv.14891

[21] Grimalt R Mengeaud V Cambazard F; Study Investigators' Group. The steroid-sparing effect of an emollient therapy in infants with atopic dermatitis: a randomized controlled study. Dermatology. 2007;214:61–7. 17191050 10.1159/000096915

[22] Szczepanowska J Reich A Szepietowski JC. Emollients improve treatment results with topical corticosteroids in childhood atopic dermatitis: a randomized comparative study. Pediatr Allergy Immunol. 2008;19:614–8. 18208463 10.1111/j.1399-3038.2007.00706.x

[23] Eberlein B Eicke C Reinhardt HW Ring J. Adjuvant treatment of atopic eczema: assessment of an emollient containing N-palmitoylethanolamine (ATOPA study). J Eur Acad Dermatol Venereol. 2008;22:73–82. 18181976 10.1111/j.1468-3083.2007.02351.x

[24] Berth-Jones J Damstra RJ Golsch S. Twice weekly fluticasone propionate added to emollient maintenance treatment to reduce risk of relapses in atopic dermatitis: randomised, double blind, parallel group study. BMJ. 2003;326:1367. 12816824 10.1136/bmj.326.7403.1367 PMC162129

[25] Zhang Y Chen Y Li C. Study on the clinical efficacy of soothing moisturizing repairing cream combined with desonide in the treatment of atopic dermatitis in children. Medicine (Baltim). 2025;104:e41277. 10.1097/MD.0000000000041277 PMC11749664 39833071

[26] Eichenfield LF Tom WL Berger TG. Guidelines of care for the management of atopic dermatitis: section 2. Management and treatment of atopic dermatitis with topical therapies. J Am Acad Dermatol. 2014;71:116–32. 24813302 10.1016/j.jaad.2014.03.023 PMC4326095

[27] Rakita U Kaundinya T Silverberg JI. Associations between shower and moisturizing practices with atopic dermatitis severity: a prospective longitudinal cohort study. Dermatitis. 2023;34:425–31. 36917546 10.1089/derm.2022.29020.jis

[28] Hon KL Pong NH Wang SS Lee VW Luk NM Leung TF. Acceptability and efficacy of an emollient containing ceramide-precursor lipids and moisturizing factors for atopic dermatitis in pediatric patients. Drugs R D. 2013;13:37–42. 23456759 10.1007/s40268-013-0004-x PMC3627015

[29] Lewis-Jones MS Finlay AY. The children’s dermatology life quality index (CDLQI): initial validation and practical use. Br J Dermatol. 1995;132:942–9. 7662573 10.1111/j.1365-2133.1995.tb16953.x

[30] European Task Force on Atopic Dermatitis. Severity scoring of atopic dermatitis: the SCORAD index. Dermatology. 1993;186:23–31. 8435513 10.1159/000247298

[31] Luger T De Raeve L Gelmetti C. Recommendations for pimecrolimus 1% cream in the treatment of mild-to-moderate atopic dermatitis: from medical needs to a new treatment algorithm. Eur J Dermatol. 2013;23:758–66. 24185493 10.1684/ejd.2013.2169

[32] Frolunde AS Thyssen JP Deleuran M Vestergaard C. Appraisal of proactive topical therapy in atopic dermatitis: pros and cons. Am J Clin Dermatol. 2021;22:775–83. 34322849 10.1007/s40257-021-00629-0

[33] Wang S Wang L Li P. The improvement of infantile atopic dermatitis during the maintenance period: a multicenter, randomized, parallel controlled clinical study of emollients in Prinsepia utilis Royle. Dermatol Ther. 2020;33:e13153. 31705602 10.1111/dth.13153

[34] Wirén K Nohlgård C Nyberg F. Treatment with a barrier-strengthening moisturizing cream delays relapse of atopic dermatitis: a prospective and randomized controlled clinical trial. J Eur Acad Dermatol Venereol. 2009;23:1267–72. 19508310 10.1111/j.1468-3083.2009.03303.x

[35] Weber TM Samarin F Babcock MJ Filbry A Rippke F. Steroid-free over-the-counter eczema skin care formulations reduce risk of flare, prolong time to flare, and reduce eczema symptoms in pediatric subjects with atopic dermatitis. J Drugs Dermatol. 2015;14:478–85. 25942666

[36] Ring J Alomar A Bieber T. Guidelines for treatment of atopic eczema (atopic dermatitis) part I. J Eur Acad Dermatol Venereol. 2012;26:1045–60. 22805051 10.1111/j.1468-3083.2012.04635.x

[37] Micali G Paterno V Cannarella R Dinotta F Lacarrubba F. Evidence-based treatment of atopic dermatitis with topical moisturizers. G Ital Dermatol Venereol. 2018;153:396–402. 29368843 10.23736/S0392-0488.18.05898-4

[38] Boonchai W Varothai S Winayanuwattikun W Phaitoonvatanakij S Chaweekulrat P Kasemsarn P. Randomized investigator-blinded comparative study of moisturizer containing 4-t-butylcyclohexanol and licochalcone a versus 0.02% triamcinolone acetonide cream in facial dermatitis. J Cosmet Dermatol. 2018;17:1130–5. 29411520 10.1111/jocd.12499

[39] Liu L Ong G. A randomized, open-label study to evaluate an intermittent dosing regimen of fluticasone propionate 0.05% cream in combination with regular emollient skin care in reducing the risk of relapse in pediatric patients with stabilized atopic dermatitis. J Dermatolog Treat. 2018;29:501–9. 29164960 10.1080/09546634.2017.1401211

[40] Hebert AA Rippke F Weber TM Nicol NH. Efficacy of nonprescription moisturizers for atopic dermatitis: an updated review of clinical evidence. Am J Clin Dermatol. 2020;21:641–55. 32524381 10.1007/s40257-020-00529-9 PMC7473959

[41] Simpson EL Chalmers JR Hanifin JM. Emollient enhancement of the skin barrier from birth offers effective atopic dermatitis prevention. J Allergy Clin Immunol. 2014;134:818–23. 25282563 10.1016/j.jaci.2014.08.005 PMC4180007

