# Supplementary material for: Efficacy of combining soothing moisturizing repairing cream with glucocorticoids in the treatment of moderate atopic dermatitis in pediatric patients: A prospective observational cohort study
Source: Medicine (Baltimore). 2026 Jan 30;105(5):e47171. doi: 10.1097/MD.0000000000047171 (PMC12863837; doi:10.1097/MD.0000000000047171)
Supplement: Supplementary file 1 [file medi-105-e47171-s001.docx]

Supplemental Table 1. Demographic and baseline disease characteristics.

|  | Experimental Group (N=29) | Control Group (N=25) | x2/t/w | P |
| --- | --- | --- | --- | --- |
| Age(years) | 7.20±1.17 | 7.5±1.17 | 1.252 | 0.358 |
| Gender,n(%) |  |  |  |  |
| Male | 13(44.83%) | 12(48%) |  | 0.816 |
| Female | 16(55.17%) | 13(52%) |  |  |
| Weight(kg) | 23.96±3.27 | 24.78±3.22 | 0.927 | 0.358 |
| Height(cm) | 125.90±4.86 | 128.88±5.65 | 1.749 | 0.086 |
| Baseline SCOARD score | 36.52±4.45 | 36.53±4.78 | 0.006 | 0.995 |
| Baseline CDLQI score | 8.05±1.93 | 8.32±2.64 | 0.638 | 0.526 |

SCOARD: Scoring of Atopic Dermatitis; CDLQI: Children’s Dermatology Life Quality Index.
